# Establishing a framework for development, prioritization, and assessment of artificial intelligence technology within the radiology department at a large UK teaching hospital

**DOI:** 10.1093/bjrai/ubag011

**Published:** 2026-05-07

**Authors:** James H R Cairns, Beverley Riley, Hanif Ismail, Bashar Al-Qaisieh, Mohua Siddique, Christopher Herbert, Bob Wheller, Fahmid U H Chowdhury, Andrew Scarsbrook

**Affiliations:** Faculty of Medicine and Health, University of Leeds, Leeds LS2 9JT, United Kingdom; Department of Radiology, Leeds Teaching Hospitals NHS Trust, Leeds, West Yorkshire, LS9 7TF, United Kingdom; Leeds Teaching Hospitals NHS Trust, Leeds, West Yorkshire, LS9 7TF, United Kingdom; Leeds Teaching Hospitals NHS Trust, Leeds, West Yorkshire, LS9 7TF, United Kingdom; Leeds Teaching Hospitals NHS Trust, Leeds, West Yorkshire, LS9 7TF, United Kingdom; Leeds Teaching Hospitals NHS Trust, Leeds, West Yorkshire, LS9 7TF, United Kingdom; Leeds Teaching Hospitals NHS Trust, Leeds, West Yorkshire, LS9 7TF, United Kingdom; Leeds Teaching Hospitals NHS Trust, Leeds, West Yorkshire, LS9 7TF, United Kingdom; Leeds Teaching Hospitals NHS Trust, Leeds, West Yorkshire, LS9 7TF, United Kingdom; Faculty of Medicine and Health, University of Leeds, Leeds LS2 9JT, United Kingdom; Leeds Teaching Hospitals NHS Trust, Leeds, West Yorkshire, LS9 7TF, United Kingdom

**Keywords:** artificial intelligence, AI, radiology, institutional approach, organizational approach, collaboration, innovation, partnership, implementation, management

## Abstract

Artificial intelligence (AI) can revolutionize clinical workflows in radiology but requires organizational change. An institutional strategy to develop and evaluate AI tools is outlined. A multidisciplinary AI board with a patient and public involvement and engagement group was created. A comprehensive framework was formed, comprising workstreams covering information governance; technical rigor, performance, and safety; economic considerations; and ethical and medical-legal aspects. In addition to recurring meetings, a workshop with clinicians, information technology specialists, and patient representatives helped to identify priority use cases. Technical infrastructure was enhanced to support the development, performance assessment, and deployment of AI tools. Primary areas for AI applications included training staff, vetting of image requests, quality assurance, image interpretation, and communicating imaging findings to patients. Potential barriers, gaps in evidence, and subsequent actions for AI implementation were outlined. Avenues for collaboration with industry and market-available solutions were outlined. A virtual Picture Archiving and Communication System server was developed and then connected to a deployment platform for performance evaluation of AI products. Establishing an institutional AI board and imaging AI sandbox has guided safe, effective AI implementation while creating an ideal setting for innovation and industry partnership. Our approach to the integration of imaging AI provides a pragmatic guide for other institutions.

## Introduction

Radiology represents an attractive opportunity for the deployment of artificial intelligence (AI) solutions given that much of its workflow is digitized. AI can play a key role in addressing the well-publicized workforce crisis affecting the National Health Service (NHS).^1^ Within the radiology department, AI can be utilized in a diverse range of applications, including vetting imaging requests, reducing radiation dose during image acquisition, prioritization of reporting, and clinical decision support. NHS England and the Department of Health and Social Care have recognized the potential to transform clinical workflow, mitigate risk, and enhance diagnostic accuracy with an investment of £250 million for the creation of the NHS AI Lab and, subsequently, £21 million for the AI diagnostic fund to accelerate the rollout of AI diagnostic technologies in Radiology.^2^ Radiology has experienced the greatest increase in AI/machine learning (ML)-enabled medical device regulatory submissions of any speciality with nearly 80% of over 1200 FDA-approved devices (as of April 2026) targeting radiology workflows.^3^ The presence of AI companies at major radiology conferences worldwide is increasing annually, but integrating AI into routine medical practice demands substantial transformation at an institutional level. This involves not only the adoption of technology but also effectively addressing the ethical, legal, and privacy challenges inherent to AI applications. Apprehension exists among some patient groups regarding the methods of AI implementation in Radiology.^4^ It is, therefore, vital that AI implementation is performed in a safe and effective manner. In this paper, we use the term AI as an umbrella term encompassing a range of computational approaches, which includes ML.^5^ We describe a method to establish a framework for development, prioritization, assessment, and patient acceptability of imaging AI at Leeds Teaching Hospitals NHS Trust (LTHT), a large teaching hospital in Yorkshire, UK.^6^^,^^7^

## Methods

Our organization established a multidisciplinary clinical AI board to navigate the complexity associated with the incorporation of AI technology in the radiology department. The key role of the board is to align, oversee, and evaluate the adoption of AI solutions and technologies related to radiological imaging. The board consists of clinical radiologists, radiographers, clinical scientists, clinicians in stakeholder specialties such as accident and emergency and acute medicine, academic and medical image analysis AI experts, trust management, information technology, and governance staff. The AI board works in partnership with a patient and public involvement and engagement (PPIE) body. This ensures patient viewpoints are considered regarding clinical implementation of AI. A holistic approach with a structured framework for AI governance is recommended to deploy and maintain AI models in clinical practice.^8^^,^^9^ The AI board operates within a structured framework comprising key workstreams such as information governance and data stewardship; integrity and performance of technical systems; financial and commercial factors; as well as ethical, legal, and privacy aspects. In addition to recurring monthly meetings with board members, a half-day workshop was conducted to highlight key priorities for implementing AI at our institution. Participants included the AI board alongside information technology technicians, radiographers, the Association of British HealthTech Industries (ABHI), and members of the PPIE group. The workshop included keynote talks from a clinical academic, a PPIE representative, and an ABHI representative to provide background and stimulate discussion, followed by small-group discussions that were subsequently fed back to the wider group. Broader group discussion was then used to identify overarching themes and key priority areas to guide subsequent product review, local research, and innovation activity. PPIE representatives helped to steer the broader group discussion by ensuring that issues of patient acceptability, transparency, trust, data use, and communication were considered alongside technical and operational priorities. PPIE involvement was then tabulated in short form according to Guidance for Reporting Involvement of Patients and the Public (GRIPP2).^10^

**Figure 1:**
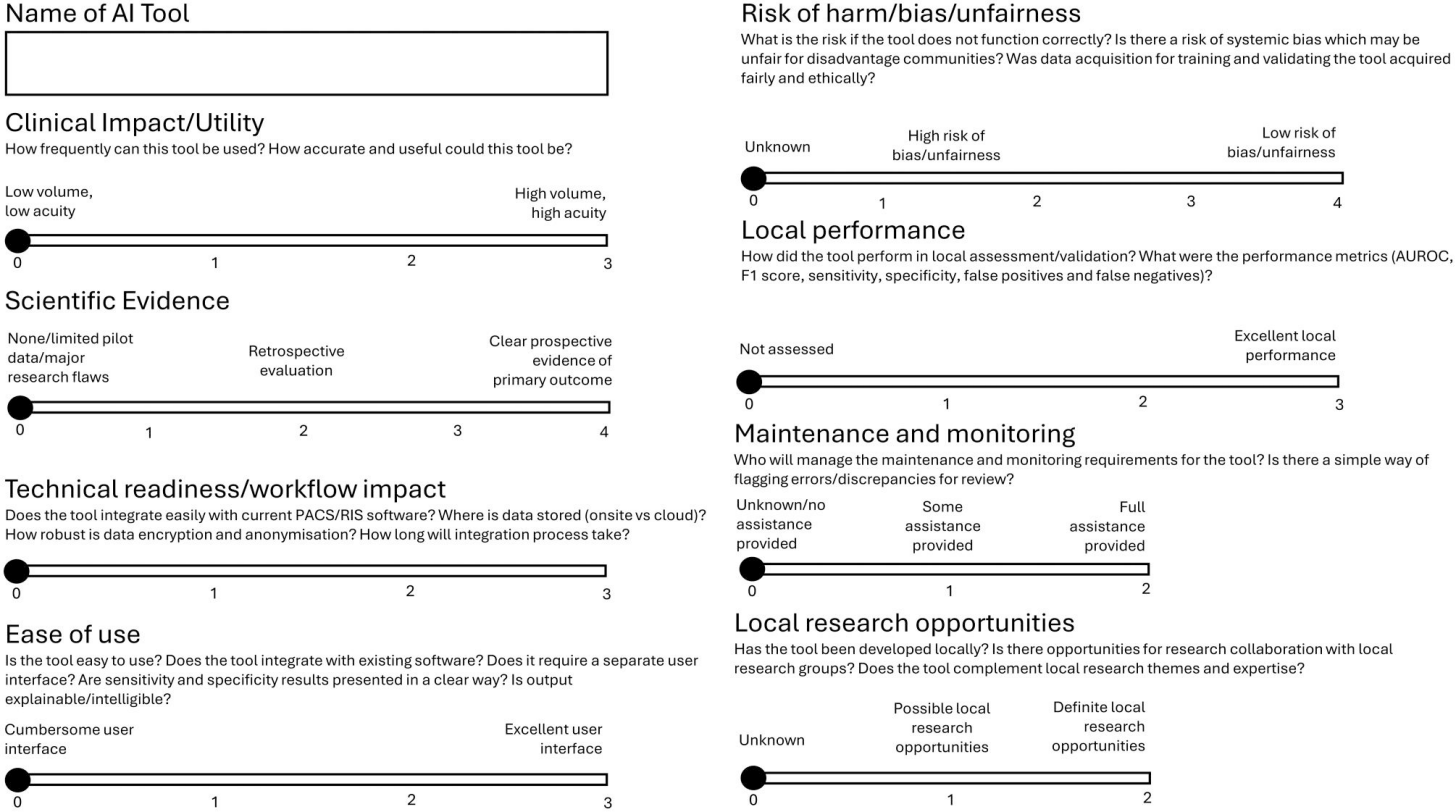
Scoring matrix used by the institutional AI board to support structured prioritization and appraisal of radiology AI products. Abbreviation: AI, Artificial intelligence. Adapted from Daye et al.^12^

There is clear guidance from the Royal College of Radiologists in the UK about how AI should integrate with the radiology reporting workflow, but at this time there is no clear guidance to support institutions with decision-making regarding which AI products to adopt.^11^ To fulfill this unmet need, a scoring matrix aligning with the AI board key work packages was modified and employed.^12^ This includes evaluation across crucial categories demonstrated in Figure 1. This aggregated score facilitates prioritization and decision-making for which AI solutions warrant more detailed evaluation. The scoring system contributes to the development of institutional memory, forming a structured accumulation of experience, rationale, and decision history that can be used to inform future AI evaluation and purchasing decisions. This is particularly beneficial in the event of workforce turnover or evolving institutional priorities.

Quantitative assessment of AI algorithms is vital and increasingly is expected to include calibration and performance monitoring over time, not only standard metrics such as sensitivity, specificity, positive predictive value, accuracy, and area under the receiver operating characteristic curve (AUROC).^13^^,^^14^ There is growing recognition that standard performance metrics such as AUROC can be misleading in imbalanced datasets. Therefore, complementary prevalence-sensitive metrics such as area under the precision-recall curve and Brier scores should be utilized for robust evaluation.^14^^,^^15^ Quantitative metrics cannot be assessed in isolation. This scoring tool ensures essential qualitative aspects are given careful consideration. For example, it assesses whether AI products meet applicable legal and regulatory requirements for medical devices (eg, holding a CE or UKCA mark and complying with the relevant UK medical device regulatory framework^16^), AI research reporting guidelines (eg, TRIPOD-AI,^17^ DECIDE-AI,^18^ CONSORT-AI^19^) and ethical principles. Ethical considerations include the potential for algorithmic bias to disproportionately affect underserved populations or exacerbate health inequalities. This is evident when training datasets are unrepresentative (eg, urban hospital bias or limited ethnicity representation).^20^^,^^21^ Multi-society consensus statements have emphasized the need for transparency, explainability, and equity when implementing AI products. They have urged AI developers and institutions to assess performance across demographic subgroups before deployment.^22^ An AI product that performs well on average may underperform in subgroups such as women, children, or ethnic minorities if these groups are underrepresented in training data. While some components of this scoring are inevitably subjective, work is ongoing to introduce standardized quantitative performance thresholds.

**Figure 2:**
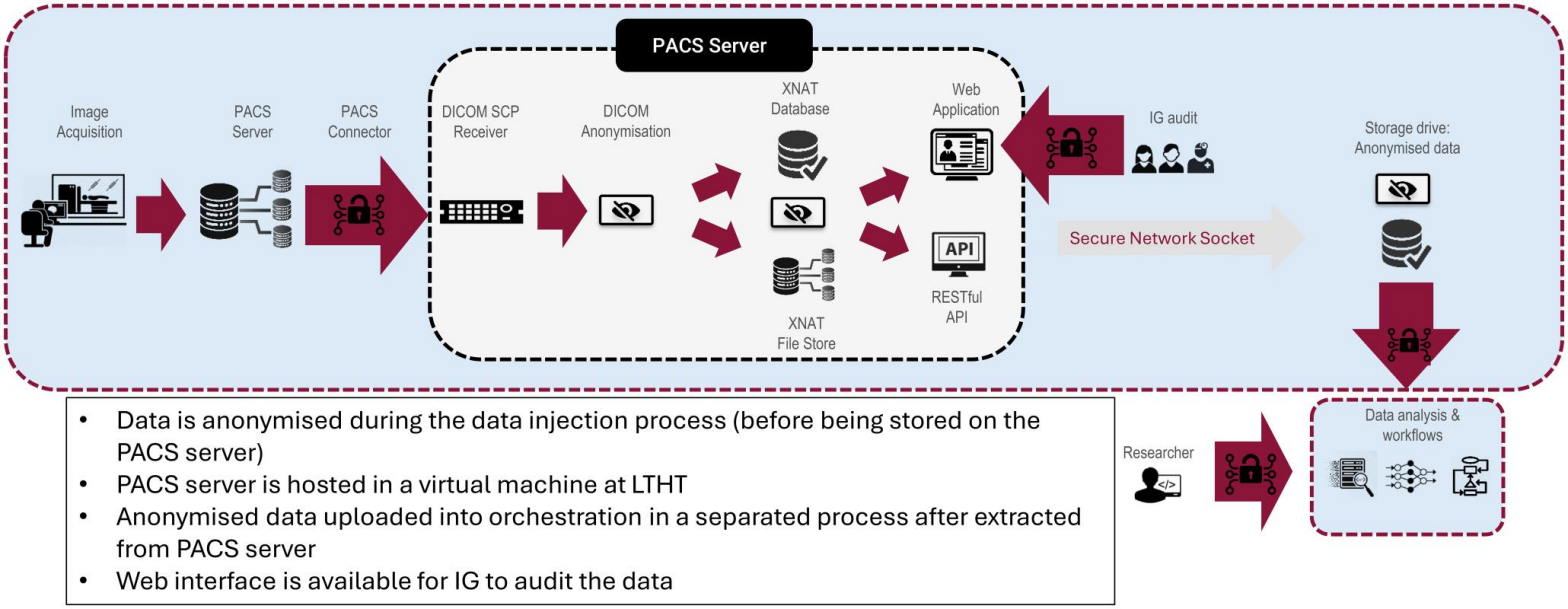
Overview of local technical infrastructure used for imaging AI development and benchmarking, including secure retrieval from institutional PACS, de-identification, storage within a controlled research environment, and monitoring. Abbreviations: API, application programming interface; DICOM, Digital Imaging and Communications in Medicine; IG, information governance; LTHT, Leeds Teaching Hospitals Trust; PACS, Picture Archiving and Communication System; SCP, Service Class Provider; XNAT, Extensible Neuroimaging Archive Toolkit.

The growth of technical infrastructure has facilitated the use of datasets to develop, validate, and assess AI products. A secure virtual Picture Archiving and Communication System (PACS) server was created. This server, together with local clinical expertise, enables the retrieval of high-quality, relevant data from the institutional PACS server (Figure 2). Data are de-identified by an embedded script within the platform server in accordance with local governance procedures and ensuring safe storage on-site. This process is managed by the institutional research data and informatics team and the data access committee and aligns with UK General Data Protection Regulation (GDPR) principles of data minimization, purpose limitation, and lawful processing.^23^ Access is controlled, and only researchers who have completed mandatory information governance training and hold valid approvals can extract de-identified data from the server to secure research environments for local analysis. An open-source vendor-neutral AI deployment engine has been implemented (https://newtonstree.ai/) linked to this server to deploy AI tools. This on-premises AI deployment engine, linked to the server, will enable performance assessment of AI software from a variety of vendors before clinical implementation in a “sandbox” environment. This will not affect the performance of clinical systems. This sandbox environment is particularly valuable as consensus on thresholds for quantitative performance metrics remains an area of unmet need and is subject to ongoing local development. The technical infrastructure will then allow integration of validated AI products from a variety of vendors into the clinical PACS system while providing essential real-time monitoring of the performance of these tools using Newton’s Tree’s Federated AI Monitoring Service (FAMOS).^24^^,^^25^

FAMOS facilitates privacy-preserving real-world concordance and calibration assessment across diverse patient subgroups. Both ‘static’ and ‘continuous learning’ radiology AI tools are prone to drift, which can degrade the performance over time and compromise patient safety. Drift is often a result of the changing environment in which the AI tools are functioning and may be due to factors such as changes in image acquisition protocols, seasonality, or patient demographics.^26^ AI products are often trained on enriched datasets, which can present issues with generalizability.^27^ FAMOS enables longitudinal monitoring of AI performance in response to changing patient demographics, imaging protocols, and epidemiological patterns such as disease seasonality or cyclicity. Recent studies have highlighted that performance degradation in real-world settings may necessitate local retraining of AI models to maintain diagnostic accuracy.^28^ However, retraining of AI models introduces complexities with regulation, as any substantive model change requires re-approval under current governance frameworks.^28^ A local implementation of FAMOS is currently underway, focusing on chest radiograph AI solutions. Every AI product implemented will undergo similar benchmarking and monitoring. Use of this sandbox and monitoring system will allow for timely and accurate identification of performance ‘drift’ to allow appropriate intervention and maintain patient safety.^13^^,^^29^^,^^30^

Productive partnership with industry has been integral to aligning AI development with targeted clinical demands and accelerating AI solution integration. New collaborations and partnerships are being established to exploit local imaging assets. Support has been provided to companies requiring datasets to develop imaging-based AI diagnostics, in line with UK GDPR principles of data minimization, secure de-identification, and clear documentation of purpose.^23^ Dataset access requests are subject to strict review protocols including data protection impact assessments and audit mechanisms. All requests are assessed for public benefit and ethicolegal alignment.

We have Health Research Authority approval to use retrospective real-world data, including data from unconsented cancer patients and patients with thoracic conditions under RCD-Onc: Enhancing understanding and prediction of cancer outcomes with baseline characteristics from routinely collected data (IRAS Ref 277122) and Thoracic-AID: Evaluation of thoracic conditions through deep learning (IRAS Ref 306378), respectively, both of which received Research Ethics Committee Proportionate Review. These approvals have covered the use of imaging datasets for development and performance benchmarking in a range of projects, following exclusion of any patients who had opted out of the use of their data.

## Results

The institutional AI board has directed the assessment of all radiological AI initiatives, delivering a consistent approach to implementing AI, which is critical to overcome common obstacles related to the implementation of AI. These obstacles include lengthy and complex information governance approvals, advancing heterogenous IT infrastructure, fostering appreciation of the impact of AI in clinical practice with patients and clinicians, and obtaining funding through partnerships with industry.^31^

**Figure 3:**
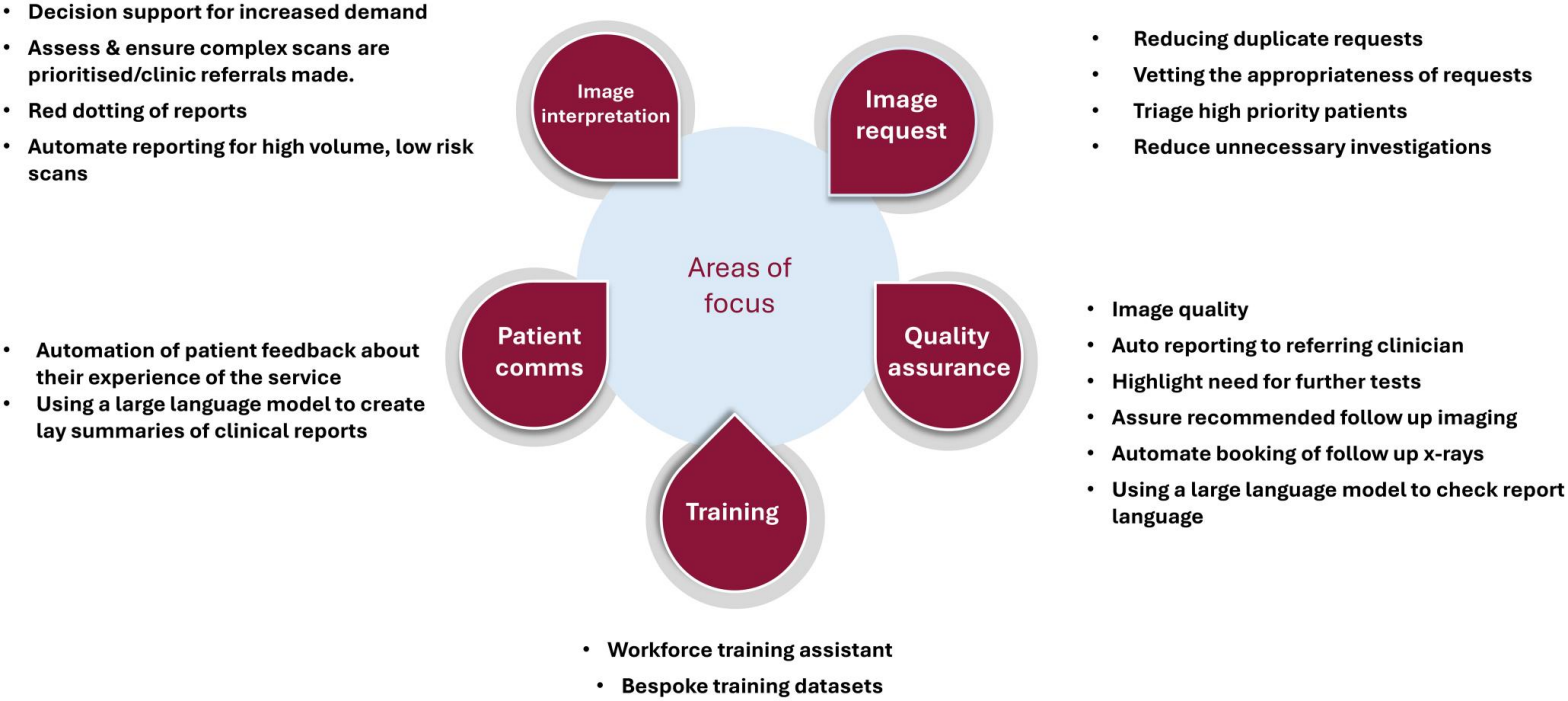
Priority domains for radiology AI implementation identified during the multidisciplinary workshop, highlighting opportunities in staff training, request vetting, quality assurance, image interpretation, and communication of imaging findings to patients.

During the prioritization workshop, key domains for applying AI were identified, such as training of staff, vetting image requests, quality assurance (monitoring of image input quality, assessing AI performance, and detecting automation bias), enhanced image interpretation, and communicating imaging findings to patients (Figure 3). Following this, a review of market-available AI products targeting the primary use cases highlighted during the workshop was performed. This process informed a “hackathon” undertaken in partnership with Leeds City Council in March 2024, in which teams of IT specialists considered a solution to a clinical problem using de-identified data. The objective of the hackathon was to explore the utility of an AI tool to simplify understanding and communication of radiology findings for patients, as no suitable commercial product was identified.

The linked PPIE group consists of 10 members with a mix of gender, age, ethnicity, religion, and lived health experience who play a multifaceted role in assisting the board. The PPIE group has offered unique perspectives on the influence of AI on the patient experience as well as prompted conversations on issues such as data ownership and value and the sharing of patient data with commercial entities. They have aided in communicating with patients regarding the use of AI technology. Through a series of co-designed workshops, representatives from the PPIE group aided in the production of educational materials including a publicly available video explaining the role of AI in healthcare (https://youtu.be/GzhmOVQ1ttY), aimed at enhancing transparency, addressing public concerns, and fostering trust through inclusive dialogue (Table 1).

**Table 1:** Guidance for Reporting Involvement of Patients and the Public Short Form (GRIPP2-SF), highlighting patient and public involvement and engagement (PPIE) in the development of the institutional radiology AI framework.^10^

Section and topic	Item
1: Aim	To develop an institutional approach to radiology AI prioritization, implementation, and monitoring, while ensuring that patient and public views informed these processes.
2: Methods	The institutional AI board worked with a linked PPI group. PPIE representatives contributed to discussions on AI implementation and took part in a half-day multidisciplinary workshop involving keynote talks, small-group discussions, and wider group feedback. They also contributed to patient-facing educational materials about AI in healthcare.
3: Results	PPIE helped ensure patient views were considered in the development of the institutional AI framework. Representatives highlighted issues including data ownership, transparency, sharing of data with commercial partners, and the need for clear communication about AI in care. A publicly available patient-facing video was produced (https://youtu.be/GzhmOVQ1ttY) to address common concerns and enhance transparency and trust in the use of AI within healthcare.
4: Discussion	PPIE broadened the focus beyond technical performance and governance to include patient trust, transparency, and acceptability. This supported a more balanced framework for AI implementation. As this was a service development project, the specific effect of PPIE on individual decisions was not quantified.
5: Reflections	Embedding PPIE within this institutional approach to AI implementation was feasible and valuable, allowing prospective consideration of patient and public concerns. The work was not designed as a formal study of PPIE impact, and therefore, the influence of PPIE was not captured using predefined process or outcome measures.

Establishing a “sandbox” equipped with an AI deployment platform enabled effective benchmarking of AI products. This environment allows assessment of AI product efficacy in enhancing efficient workflow and performance in diagnosis before full implementation in clinical practice. There is potential for a substantial performance gap between legal certification and real-world clinical efficacy.^32^ This gap arises because regulatory approval requires only limited evidence of efficacy, such as internal validation studies. Real-world performance depends on broader contextual factors, such as imaging protocols and epidemiological patterns, as previously described. Van Leeuwen et al highlighted in a study of 100 commercially available AI tools with CE marking that 64 of these lacked peer-reviewed evidence, and only 18 demonstrated a tangible clinical impact.^32^

The partnerships formed with industry collaborators assist in leveraging AI to enhance the radiology department and improve patient care. A collaboration with Lucida Medical (https://lucidamedical.com) has been established under a Topol Fellowship feasibility project to evaluate the use of AI to triage patients with prostate cancer. The institution has been awarded seed funding from the Yorkshire Imaging Collaborative to facilitate the evaluation of 2 radiology AI products for body computed tomography and musculoskeletal plain radiographs. A collaboration with Mirada Medical (https://www.mirada-medical.com) has also been funded by Innovate UK. A lung cancer prediction AI algorithm developed by Optellum (https://optellum.com) has been deployed and is being evaluated in a prospective non-interventional real-world study. Collaborations such as these have resulted in academic output supported by industry resources. An important result of this cooperation is the creation of AI technologies that are specifically tailored for real-world radiological applications. A prime example of this is the development of AI-supported interpretation of PET-CT in assessing response to treatment in patients with lymphoma. This has demonstrated promise in decreasing the time required for interpretation while maintaining accuracy.^33^

## Discussion

AI is an area of intense imaging research activity and commercial development that currently has limited clinical use in most settings in the UK. Establishment of an institutional AI board, a linked PPIE group, and a virtual “sandbox” infrastructure with an AI deployment platform has supported the safe and effective implementation of AI in the radiology department at our institution. This framework is particularly important in mitigating safety concerns such as performance drift, subgroup underperformance, and automation bias. Ethical and legal considerations such as transparency, fairness, and compliance with UK GDPR are actively incorporated into our assessment of AI technology, and there is ongoing PPIE engagement.

This institutional approach has several benefits for key stakeholders. Clinicians are provided access to AI-supported decision-making, patients are actively involved through co-produced educational content, industry partners are guided by a single point of access to facilitate effective GDPR-compliant dataset usage, and IT teams benefit from streamlined infrastructure aligned with deployment needs. Tangible benefits include reduced time-to-deployment for approved AI tools, successful funding bids for AI deployment, and improved prioritization of relevant commercial AI solutions for clinical implementation. Intangible benefits include increased institutional trust, workforce upskilling, and strengthened academic-industry relationships.

Some basic health economic considerations have been integrated into our assessments in the form of net cost. Formal health technology assessments (HTAs)^34^^,^^35^ are time- and resource-intensive, and more pragmatic frameworks such as return on investment (ROI) assessments may be more suitable. ROI assessments include metrics such as diagnostic throughput and avoided downstream costs due to improved triage or earlier detection of disease, which provide tangible cost savings to institutions.^36^

There is currently no established framework for legal liability when AI contributes to diagnostic error. While CE/UKCA marking approves conformity to a certain standard, our board’s assessment and local validation processes aid in clarification of responsibility to ensure safe deployment in our clinical context. Additionally, we plan to place emphasis on selecting AI products with explainability features to allow interrogation of output by clinicians to support informed decision-making and clear accountability.

A limitation of this project has been the allocation of IT resources, which can hamper progress despite strong clinical support. This institutional approach with the establishment of the AI board has facilitated collaboration among healthcare professionals to devise measures that optimize the distribution of limited resources and address this issue. This has also opened lines of communication to colleagues in support services such as scientific computing.

Future planned work includes local implementation of the FAMOS framework supporting real-world performance monitoring and fairness auditing across important patient subgroups. Our institution is also undertaking an AI deployment fund study evaluating a commercial chest x-ray tool. Further publications are planned to disseminate these findings.

These measures will facilitate ongoing innovation collaboration with industry, in addition to ongoing enhancements in clinical care and radiology department operations. The AI board is set to be pivotal in the provision of AI-integrated services at our large teaching hospital.
